# Genetic potential and inheritance pattern of agronomic traits in faba bean under free and infested *Orobanche* soil conditions

**DOI:** 10.1186/s12870-024-05017-4

**Published:** 2024-04-19

**Authors:** Alaa A. Soliman, Mohamed A. Ibrahim, Manar I. Mousa, Elsayed Mansour, Yuhua He, Haitian Yu

**Affiliations:** 1 Food Legumes Research Department, Agricultural Research Center, Field Crops Research Institute, Giza, 12619 Egypt; 2 Institute of Food Crops, Yunnan Academy of Agricultural Sciences, Kunming, 650205 China; 3 Department of Crop Science, Faculty of Agriculture, Zagazig University, Zagazig, 44519 Egypt

**Keywords:** Biotic stress, Breeding, Combining ability, Faba bean, GGE biplot, Heterosis, line×tester, *Orobanche*

## Abstract

**Background:**

*Orobanche* is an obligate parasite on faba bean in the Mediterranean region, causes considerable yield losses. Breeding tolerant faba bean genotypes to *Orobanche* is pivotal to sustain production and ensuring global food security, particularly considering the challenges posed by population growth. In the present study, seven faba bean lines and four testers were used in a line×tester mating design during 2020–2021 and 2021–2022 growing seasons. The eleven parents and their 28 F_1_ crosses were evaluated under *Orobanche* free and naturally infested soils.

**Results:**

The results demonstrated considerable variations among the evaluated genotypes, wide diversity among the parental materials, and heterotic effects for all studied agronomic traits under *Orobanche*-free and infested soils. *Orbanche* infestation displayed a significant adverse impact on all the studied agronomic traits. The genotypes Line1, Line2, Line3, and Line5 displayed superior performance under *Orobanche*-infested conditions and recorded the highest values of all studied agronomic traits. Additionally, Line1, Line2, Line3, Line5, and Line7 exhibited desirable significant GCA for most evaluated traits under the two infestation conditions. The obtained crosses displayed significant negative or positive heterosis for studied agronomic characters such as plant height, number of branches per plant, number of pods per plant, number of seeds per plant^,^ and seed weight per plant were observed. Furthermore, specific cross combinations such as Line2×Sakha3, Line3×Nubaria5, Line7 × Nubaria5, Line6×Nubaria1, Line5×Sakha3, Line1×Sakha3, and Line1 × Nubaria5 exhibited superior performance in seed yield and contributing traits under *Orobanche*-infested conditions. Moreover, these specific crosses showed superior efficacy in reducing dry weight of *Orobanche* spikes. The results obtained from GGE biplot analysis closely aligned with those from the line×tester procedure, affirming the significance of GGE biplot as a valuable statistical tool for assessing genotype combining ability in line× tester data. Both additive and non-additive gene actions were reported to be predominantly involved in the inheritance of the studied agronomic traits in faba bean.

**Conclusions:**

The detected genetic diversity within the evaluated faba bean genotypes and their developed crosses exhibits substantial potential for improving faba bean productivity under *Orobanche*-infested conditions. The parental genotypes, Line1, Line2, Line3, Line5, and Line7, were identified as effective and promising combiners. Moreover, the developed crosses Line2×Sakha3, Line3×Nubaria5, Line7×Nubaria5, Line6×Nubaria1, Line5×Sakha3, Line1×Sakha3, and Line1×Nubaria5 could be considered valuable candidates for developing high-yielding and tolerant faba bean genotypes to *Orobanche*.

**Supplementary Information:**

The online version contains supplementary material available at 10.1186/s12870-024-05017-4.

## Background

Faba bean (*Vicia faba*) is the primary pulse crop serving as a pivotal protein source for both human and animal nutrition [1]. Its cultivation within crop rotation contributes significantly to nitrogen fixation, crop diversification and soil biodiversity [2]. Globally, its cultivated area is approximately 35 × 10^6^ hectares yielding an annual production of about 27.7 × 10^6^ tons [3]. Egypt contributes to these figures with 42 × 10^3^ hectares of cultivation and a production of 139 × 10^3^ tons [3]. However, faba bean production is decreasing annually in Egypt creating a widening gap between local production and consumption due to economic factors, population growth, and abrupt climate fluctuations [4]. Its susceptibility to yield instabilities remains a considerable obstacle influenced by various reasons including diseases, pests, *Orobanche* infestations, and less favorable environmental conditions [5]. Addressing these challenges has become imperative to diminish the gap between consumption and production to enhance global food security.

*Orobanche crenata* is an annual parasitic plant widespread across the Mediterranean region, West Asia, and North and East Africa [6]. It relies entirely on the host due to chlorophyll shortage. It attaches to host roots and survives by absorbing carbohydrates from phloem and water along with elements from the xylem through a bridge to vascular tissues [7]. Upon infection, *Orobanche* utilizes the plants for water, nutrients, metabolites, and hormones. It leads to a considerable reduction in the number of host flowers, shedding of pollinated flowers, premature fruit drops, or suboptimal fruit growth [8]. Accordingly, this parasitic plant poses a severe threat to faba bean causing yield losses of up to 80% [9]. These losses vary based on factors including environmental factors, soil moisture, sowing date, parasitism level, and host genotype [10]. Environmental factors, particularly temperature, play a significant role in *Orobanche* development. Higher temperatures are often linked to increased *Orobanche* parasitism, while lower temperatures correlate with reduced infections. Additionally, water availability may influence *Orobanche* development [11]. Moreover, faba bean cultivars exhibit varying levels of tolerance to *Orobanche crenata.* Inherent genetic tolerance to *Orobanche* is influenced by genetic factors. Hence, developing tolerant genotypes could offer reliable defense against *Orobanche*, potentially improving faba bean yield and stability [12].

Tolerance mechanisms against *Orobanche* can be attributed to specific traits that inhibit germination, hinder the attachment, penetration, nutrient extraction, or establishment of the parasitic weed on the host plant. Agronomic traits that contribute to the tolerance of crops to *Orobanche*, a parasitic weed, involve characteristics that either minimize the impact of *Orobanche* infestation or enhance the crop ability to withstand and recover from the parasitic attack. Maalouf et al. [13] demonstrated significant variations among faba bean genotypes in *Orobanche* plant number, dry weight, *Orobanche* index, flowering date, maturing date, and seed yield. Certain newly developed lines exhibited satisfactory yield stability in the presence of *Orobanche* infestation when compared to susceptible genotypes. In soils with high infestation levels, tolerant and resistant genotypes displayed acceptable seed yield in comparison to the susceptible genotypes. However, the yield potential of resistant and tolerant lines in non-infested soils was found to be lower than that of the highly susceptible check. Moreover, Rubiales et al. [14] elucidated that seed yield was adversely impacted, primarily by *Orobanche* infection, followed by ascochyta blight and chocolate spot infection with a lesser effect. Furthermore, specific faba bean genotypes were identified as promising candidates for cultivation in the region, suggesting their potential for integration into future breeding programs.

Success in plant breeding programs depends on identifying promising parents with high yields and robust tolerance to biotic and abiotic stress factors [15–17]. Combining ability analysis serves as a valuable tool to select parents based on their cross-performance, aiming to identify superior combiners [18, 19]. This analysis is crucial in exploiting heterosis and developing favorable and heritable genes. The line×tester analysis can be employed to estimate general and specific combining abilities across various traits of faba bean [20–22]. Improvement of yield traits resulting from heterozygosity due to outcrossing has been extensively documented in faba bean [23, 24]. Heterosis, derived from the interaction between allelic and interallelic genes, offers effective improvement in agronomic traits. Utilizing heterosis through crosses can substantially augment and stabilize faba bean yields [25–27]. Moreover, it becomes imperative to comprehensively explore the inheritance nature and heritability of significant characteristics. The genetic basis of crucial agronomic traits in faba bean, under both *Orobanche*-free and infested soils, were significantly affected by non-additive and additive gene actions [28, 29].

Breeding designs face challenges in result visualization despite their diverse applications. The biplot approach developed for the analysis of combining abilities, heterosis, and parent relationships, employs principal components (PC1 and PC2) obtained through principal component analysis for graphical data representation [30]. The genotype plus genotype by environment (GGE) biplot is applied for studying GCA and SCA in a cross and aids in identifying superior cross combinations for line×tester data [31–33]. The present study aimed at (i) developing desirable general and specific combiners for developing high-yielding and tolerant faba bean genotypes to *Orobanche*, (ii) assessing inheritance patterns for agronomic traits of faba bean, (iii) utilizing visualization GGE biplot to estimate optimal parents and crosses, and (4) identifying superior cross combinations for further utilization in faba bean breeding programs focused on *Orobanche* tolerance.

## Materials and methods

### Experimental site and plant material

This work was performed at the Experimental Farm of Sakha Research Station (30°56′ N and 31°05′ E) during 2020–2021 and 2021–2022 winter seasons. The climatic data for the two growing seasons are outlined in Table S1. Additionally, the soil properties of the experimental site are detailed in Table S2. Eleven faba bean parents that used in this study were obtained from the Legumes Research Department, Agricultural Research Center, Egypt. All genotypes complied with national, international, and institutional legislation and guidelines. Table 1 provides names, pedigrees, and *Orobanche* reactions of the lines and testers used in this study. The used testers are high-yielding commercial cultivars while susceptible to *Orobanche*, in contrast, the employed seven lines are tolerant *Orobanche*. In 2020–2021 season, the lines and testers were crossed to produce the cross seeds of 28 F_1_ crosses by applying the line×tester mating design method. In 2021–2022 season, the 28 F_1_’s and their eleven parents were assessed in a randomized complete block design with three replicates. The studied genotypes were evaluated separately under both *Orobanche*-free and naturally-infested soils. Two fields of *Orobanche*-free and *Orobanche*-infested are designated for assessing faba bean genotypes at Sakha Research Station. All agricultural practices were applied as recommended except for *Orobanche*. Two ridges represented the parents and their F_1_s. Seeds were planted on ridges, each ridge was 3-m in length and spaced 0.6-m apart. Single-seeded hills were positioned along one side of the ridge, maintaining a distance of 0.20-m between hills.

**Table 1:** Name, pedigree, reaction to *Orobanche* and seed type of the evaluated faba bean parental lines and testers

Genotypes	Pedigree	Reaction to *Orobanche*	Seed type
**Lines**			
Line1	Sakha1×Misr 1	Tolerant	Equina
Line2	Sakha5	Tolerant	Equina
Line3	Giza 843×Misr 3	Tolerant	Equina
Line4	Nubaria3×Misr 1	Tolerant	Equina
Line5	H 2124/99	Tolerant	Equina
Line6	Misr 3×H 1907	Tolerant	Equina
Line7	H 2097	Tolerant	Equina
**Tester**			
Nubaria1	Individual plants selected from the Spanish variety	Susceptible	Major
Sakha3	Giza461 × 503/453/83	Susceptible	Equina
Nubaria5	landraces of Hamam 10	Susceptible	Equina
Marina	*Vicia faba* L.	Susceptible	Minor

### Data collection

Data were recorded on 15 guarded plants for each genotype. The studied traits included plant height (cm), number of branches per plant, number of pods per plant, number of seeds per plant, and seed yield per plant (g) under the two types of fields (*Orobanche* free and infested fields). In addition, dry weight of *Orobanche* spikes per plot (g) were recorded under *Orobanche*-infested soil only at harvest.

### Stress susceptibility index

A stress susceptibility index was utilized to describe the relative tolerance of the evaluated genotypes under infested field with *Orobanche* [34]. High values of the stress susceptibility index indicate susceptibility to *Orobanche*, while low values suggest tolerance in the assessed genotypes. The stress susceptibility index was estimated as (1 – s/ n)/ D, Where SI = an index of *Orobanche* susceptibility, s = genotype mean under *Orobanche* stress condition, n = genotype mean under free-*Orobanche* soil, D = Environmental stress intensity = 1 – (mean of all evaluated genotypes under infested-*Orobanche* soil/ mean of all genotypes under Free-*Orobanche* soil).

### Statistical analysis

Plot averages were used to analyze the tested traits using the regular analysis of variance for randomized complete block design in each experiment separately. Genotype variances were partitioned into parents, crosses, and parents vs. crosses (heterosis). The significance of difference among averages was tested using the least significant difference (LSD) at both 5% probability levels. The line×tester analysis was done in case of significant differences among genotypes to explore general and specific combining abilities according to [20] and described by [35]. The proportional contribution of lines (females), testers (males), and their interactions to total variance were computed. The heterosis as the percentage deviation of F_1_ mean performance from mid-parent was estimated according to [36]. Genotype plus genotype by environment biplot was employed to analyze two-way data in which rows and columns represent different experimental units. for line×tester data, the row is considered as a “line” and the column as a “tester” [30]. The mathematical model for the GGE biplot analysis of the line×tester data has been explained by [37]. Data were analyzed using Genstat software.

## Results

### Analysis of variance

The analysis of variance for the examined traits under *Orobanche* soil treatments, including genotypes, parents, crosses, lines, testers, and their interactions, are illustrated in Table 2. The mean squares of the soil treatments were significant at probability level 0.05 or 0.01 for all traits. Meanwhile, mean squares of genotypes were significant for all examined traits under all conditions. The variations due to parent crosses and parent vs. crosses were significant for all traits. In addition, the variations due lines were significant for all traits, except for plant height and number of branches/plant in all conditions, number of seeds per plant under infested soils, seed weight/plant under infested and combined conditions, and dryweight of *Orobanche* spikes under infested soils. The mean squares of testers were significant for all traits except for number of pods/plant under free soils, seed weight/plant under free soils, and dry weight of *Orobanche* spikes under infested soils. Moreover, lines×testers mean squares were significant in all cases. The mean squares due to soil treatment interactions with all sources of variations were significant for all traits, except for interaction with lines for all traits and interactions with testers for number of branches per plant and seed weight/plant.

**Table 2:** Analysis of variance (mean squares are presented) for the studied traits of assessed genotypes under both *Orobanche*-free and infested soil conditions

**Source of variance**	**df**	**Plant height (cm)**		**No. of branches** **per plant**		**No. of pods** **per plant**		
		***O.*-Free**	***O.*-Infested**	***O.*-Free**	***O.*-Infested**	***O.*-Free**	***O.*-Infested**	
Genotype	38	178.4 ^**^	1325 ^**^	3.80 ^**^	2.30 ^**^	275.5 ^**^	125.4 ^**^	
Parent	10	186.1 ^**^	2237 ^**^	3.90 ^**^	1.40 ^**^	144.7 ^**^	150.6 ^**^	
Parent vs. Cross	3419 ^**^	6583^**^	65.30 ^**^	18.30^*^	4182 ^**^	108.2 ^*^		
Cross	27	55.50 ^**^	792.2 ^**^	1.50 ^**^	2.10 ^**^	179.3 ^**^	116.7 ^**^	
Line	6	26.70	797.6	0.50	2.60	502.0 ^**^	232.1 ^*^	
Tester	203.2 ^*^	2656 ^**^	7.10 ^**^	6.50 ^**^	116.6	219.6 ^*^		
Line×Tester	18	40.50 ^**^	479.7 ^**^	0.90 ^**^	1.10 ^**^	82.20 ^**^	61.00 ^**^	
Error	76	1.00	1.50	0.10	0.20	8.10	3.40	
CV		0.80	1.30	7.40	10.10	6.90	11.50	
**Source of variance**	**df**	**No. of seeds** **per plant**		**Seed weight** **per plant (g)**		**Dry weight of *Orobanche *spikes/plot (g)**		
		***O.*-Free**	***O.*-Infested**	***O.*-Free**	***O.*-Infested**	***O.*-Infested**		
Genotype	38	1781 ^**^	1374 ^**^	953.7 ^**^	821.0^**^	32,379 ^**^		
Parent	10	1833 ^**^	1647 ^**^	786.6 ^**^	824.9^**^	50,962 ^**^		
Parent vs. Cross	1	34,206 ^**^	702.0 ^**^	19,036 ^**^	723.9^*^	8064.7^**^		
Cross	27	561.0 ^**^	1297 ^**^	345.9 ^**^	823.1^**^	26,396^**^		
Line	6	1392 ^**^	1963	715.8 ^*^	810.8	35,999		
Tester	3	1062 ^**^	2820 ^*^	356.5	2187^*^	36,968		
Line×Tester	18	200.7 ^**^	821.7 ^**^	220.9 ^**^	599.9^**^	21,435^**^		
Error	76	10.80	10.0	14.60	7.20	40.10		
CV		2.60	8.70	4.30	9.40	3.10		

* and ** signify *P* value < 0.05 and 0.01, in the same order

### Mean performance

The mean performance of the studied traits for lines, testers, and their F_1_ crosses is presented in Table 3. Plant height decreased significantly by 21.9% under *Orobanche*-infested soil. The reduction percentage due to *Orobanche* infection ranged from 0.22 to 17.6% in lines, 49.4 to 64.5% in testers, and 0.01 to 49.2% in the developed crosses. The shortest height was recorded by Line3, and specifically by Line6 and Line7. Meanwhile, the tallest plants were assigned for Line1 under *Orobanche*-free and infested soils. The shortest crosses were Line3×Sakha3 and Line5×Marina, while Line1×Marina and Line2×Sakha3 were the tallest ones under *Orobanche*-free and infested soils, respectively.

Number of branches per plant decreased significantly by 39.75% reduction under *Orobanche*-infested soil. The reduction percentage ranged from 2.2 to 50.9% in lines, 13.5 to 73.6% in testers, and 16.7 to 68.4% in crosses. Under *Orobanche*-free soil, the highest number of branches/plant belonged to Line4, while Line3 produced the lowest number (Table 3). On the other hand, under *Orobanche*-infested soil, Line1 produced the superior number of branches per plant^,^ and Line5 gave the lowest number. The highest number of branches per plant was observed by the cross combination Line4×Nubaria1, while the minimal values were recorded by Line2×Marina and Line6×Marina under *Orobanche*-free and infested soils, respectively.

Number of pods per plant decreased significantly by 71.10% under *Orobanche*-infested soil. The reduction percentage ranged from 31.2 to 80.9% in lines, 82.2 to 90.8% in testers, and 41.3 to 92.2% in crosses. The number of pods/plant ranged from 24.9 to 7.2 pods in Line7 and Line6 to 47.00 and 22.81 pods in Line1 and Line3 (Table 3). The range of testers was 22.8 and 2.1 Nubaria1 to 31.20 and 5.54 in Sakha3 under *Orobanche*-free and infested soils, respectively. The crosses ranged from 31.8 to 13.3 pods in Line7×Nubaria5 and Line7×Nubaria1 to 66.3 and 26.3 in Line1×Marina and Line2×Sakha3 under *Orobanche* free and infested soils, respectively.

Number of seeds per plant significantly decreased under *Orobanche*-infested soil compared to free soil by 70.84%. The decreasing percentages ranged from 33.8 to 79.4 in lines, 76.0 to 94.0 in testers, and 41.8 to 93.8% in crosses. The highest seed number was recorded in Line1 and Line3, and the lowest numbers were observed in Line7 (Table 3). While seed number in testers ranged from 46.8 to 35.0 seeds in Marina and Nubaria1 to 108.9 and 22.7 seeds in Nubaria5 and Sakha3 under *Orobanche*-free and infested soils, respectively. The highest seed numbers were detected by Line6×Nubaria1 and Line7×Nubaria1, whereas the lowest values were obtained by Line1×Marina and Line2×Sakha3 under *Orobanche*-free and infested soils, respectively.

Seed yield per plant was decreased significantly by 68.2% under *Orobanche*-infested soil. The reduction percentage ranged from 30.9 to 71.3 in lines, 78.1 to 94.6 in testers, and 37.4 to 94.0 in crosses. The superior seed yield per plant in lines was recorded by Line1 and Line5, while the lowest values belonged to Line4 under *Orobanche*-free and infested soils (Table 3). The seed weight of testers ranged from 27.0 to 3.9 g in Marina to 85.5 and 18.7 g in Sakha3 under *Orobanche*-free and infested soils, respectively. The greatest seed yield per plant was produced by crosses Line7×Nubaria1 and Line2×Sakha3, while the lowest values belonged to the crosses Line4×Nubariaand Line7×Marina under *Orobanche*-free and infested soils.

The lowest dry weight of *Orobanche* spikes/plot (g) was recorded by Line3, while Line5 showed the highest weight. The tester Marina showed the lowest weight, while Nubaria5 gave the highest weight. The crosses, Line2×Sakha3 exhibited the lowest dry weight of *Orobanche* spikes followed by Line1×Sakha3 and Line5×Marina. Otherwise, Line5×Nubaria5 followed by Line6×Sakha3, Line2×Nubaria1, and Line5×Nubaria1displayed the highest dry weight of *Orobanche* spikes/plot (g).

### Stress susceptibility index

**Fig. 1:**
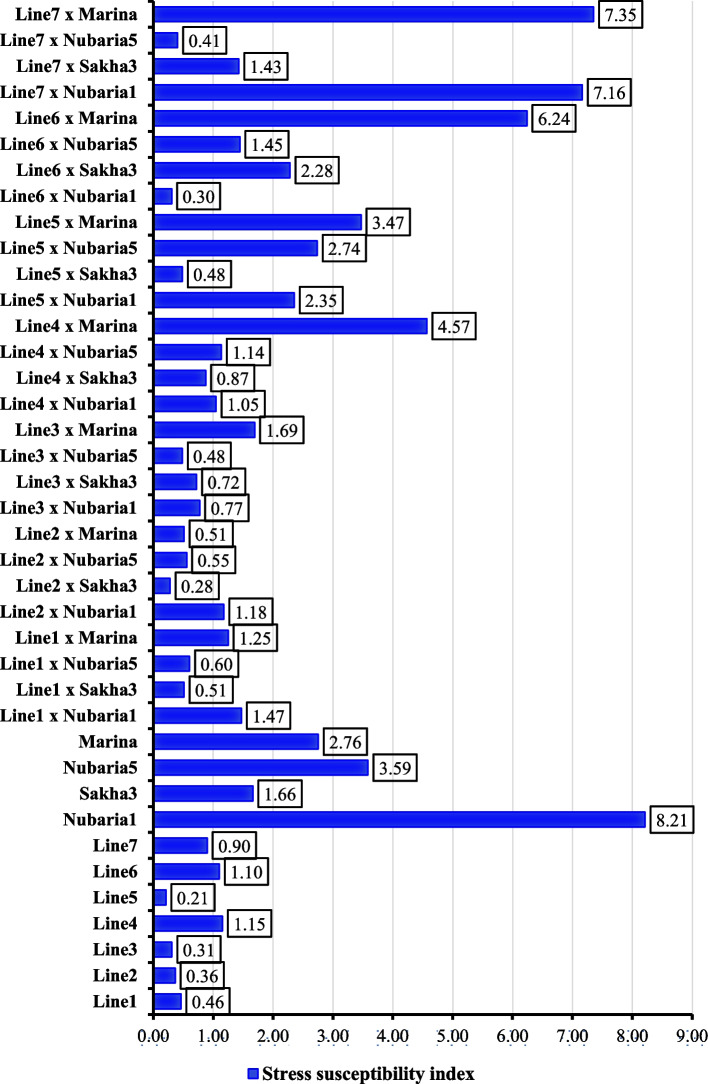
Stress susceptibility index based on seed yield per plant for evaluated parental genotypes and their crosses

The results of stress susceptibility index based on seed yield indicated that the lines were tolerant or moderately tolerant to *Orobanche* particularly Line5 was the most tolerant genotype followed by Line3, Line2, and Line1 (Fig. 1). On the other hand, testers were susceptible to *Orobanche*, particularly Nubaria1 was the most susceptible genotype followed by Nubaria5 and Marina. The most tolerant crosses were Line2×Sakha3, Line6×Nubaria1, Line7×Nubaria5, Line3×Nubaria5, Line5×Sakha3, Line2×Marina, Line1×Sakha3, Line2×Nubaria5, Line1×Nubaria5, Line3×Sakha3, Line3×Nubaria1 and Line4×Sakha3. The aforementioned crosses had values of SSI less than one. Otherwise, the most susceptible crosses were Line7×Marina, Line7×Nubaria1, Line6×Marina, Line4×Marina and Line5×Marina.

**Table 3:** Mean performance of the studied agronomic traits for parental lines and testers, and their crosses under both *Orobanche*-free and infested soils conditions

Genotypes	Plant height (cm)		No. of branches		No. of pods per plant			No. of seeds per plant			Seed weight per plant (g)	Dry Weight of Orobanche spikes/plot (g)
	*O.* Free	*O.* Infested	*O*. Free	*O*. Infested	*O*. Free	*O*. Infested	*O*. Free	*O*. Infested		*O*. Free	*O*. Infested	
**Lines**												
Line1	119.4	114.7	4.25	3.22	47.00	16.90	132.58	56.57	78.95		39.74	214.8
Line2	114.2	103.4	3.83	2.38	30.33	17.88	96.50	55.88	68.54		38.46	284.8
Line3	106.0	104.1	3.00	2.94	33.14	22.81	130.27	69.69	77.10		46.35	199.6
Line4	114.3	111.0	4.43	2.67	34.06	10.34	112.29	28.08	61.43		17.66	334.8
Line5	109.3	109.1	3.71	1.82	33.29	16.80	90.12	59.68	73.03		50.42	423.6
Line6	107.5	88.57	3.75	2.72	37.50	7.17	104.88	21.59	70.14		20.85	380.1
Line7	107.1	88.33	3.52	2.17	24.86	7.67	73.27	20.33	61.63		21.01	244.1
**Testers**												
Nubaria1	118.3	50.22	6.00	3.11	22.83	2.11	83.83	5.03	83.93		4.51	60.6
Sakha3	107.9	47.72	5.86	1.54	31.20	5.54	94.54	22.66	85.54		18.73	85.1
Nubaria5	123.7	62.59	4.10	3.54	30.57	3.54	108.85	11.30	77.47		8.91	111.1
Marina	130.3	46.22	2.00	1.61	23.00	3.21	46.83	6.93	27.02		3.91	49.6
**Crosses**												
Line1×Nubaria1	122.5	100.3	6.22	3.08	51.83	10.18	140.67	29.30	99.33		23.89	253.5
Line1×Sakha3	120.8	119.0	6.04	3.50	54.63	19.64	156.38	66.56	101.45		48.31	81.4
Line1×Nubaria5	128.7	115.3	6.63	4.00	59.88	17.44	168.00	59.61	105.63		45.97	126.3
Line1×Marina	135.2	112.7	5.00	3.14	66.33	14.14	173.50	40.67	98.93		26.88	105.0
Line2×Nubaria1	120.0	96.83	6.57	3.72	51.60	12.33	121.60	34.61	94.16		26.71	320.3
Line2×Sakha3	123.3	123.3	6.19	5.06	44.72	26.25	141.05	82.04	98.63		61.72	78.70
Line2×Nubaria5	127.1	110.7	5.91	3.72	38.02	20.20	127.40	59.55	87.31		39.87	140.3
Line2×Marina	134.3	106.4	4.04	3.08	44.89	18.89	135.64	57.92	80.77		38.46	101.0
Line3×Nubaria1	120.1	110.3	5.69	4.43	38.90	13.89	136.66	31.32	102.81		38.62	255.0
Line3×Sakha3	118.6	104.3	5.90	4.03	45.57	17.70	131.38	46.84	104.62		41.04	231.2
Line3×Nubaria5	130.6	110.1	5.56	4.11	51.94	23.86	140.63	74.40	110.52		54.41	141.7
Line3×Marina	128.2	75.28	5.11	2.58	48.44	8.46	130.89	25.80	86.72		18.71	169.0
Line4×Nubaria1	122.4	115.1	6.64	5.53	43.76	12.50	128.95	35.38	92.73		28.57	159.5
Line4×Sakha3	124.5	117.6	5.51	3.87	49.95	13.50	135.77	42.38	92.56		32.17	167.3
Line4×Nubaria5	127.4	111.6	5.49	3.04	46.02	10.80	126.68	32.91	77.46		22.54	204.3
Line4×Marina	124.4	75.3	5.00	2.64	39.83	4.64	139.83	12.92	84.30		7.80	136.3
Line5×Nubaria1	130.5	105.3	6.71	3.00	37.86	6.30	123.67	19.00	103.32		17.09	310.2
Line5×Sakha3	125.9	115.0	5.45	3.70	35.86	17.98	132.28	63.08	103.81		50.99	260.1
Line5×Nubaria5	124.7	86.97	5.46	3.09	42.19	6.11	126.80	15.64	85.66		12.46	497.7
Line5×Marina	128.7	65.35	6.27	1.98	49.73	7.94	145.06	22.62	108.16		12.80	161.3
Line6×Nubaria1	122.5	110.8	5.98	3.33	38.73	16.33	111.84	56.50	85.96		52.06	137.7
Line6×Sakha3	125.1	83.78	6.45	2.72	41.89	7.07	134.21	20.74	103.61		17.57	327.4
Line6×Nubaria5	126.2	110.0	5.09	3.00	38.49	8.75	129.73	25.25	95.89		23.36	260.3
Line6×Marina	132.5	70.35	4.83	1.55	41.17	4.61	138.83	10.83	96.82		6.73	151.3
Line7×Nubaria1	131.1	83.62	6.32	3.06	36.93	3.29	129.27	8.88	129.46		7.90	292.8
Line7×Sakha3	127.4	100.3	5.97	3.29	38.76	8.31	126.86	27.89	107.28		26.41	169.4
Line7×Nubaria5	126.9	100.3	5.33	3.90	31.83	15.14	115.75	51.17	101.75		54.40	116.7
Line7×Marina	129.1	85.38	4.25	3.05	47.50	3.70	148.35	9.20	100.69		6.00	207.0
Mean	123.0	96.08	5.23	3.15	41.16	11.89	124.91	36.43	89.87		28.56	203.9
LSD_0.05_	1.60	2.02	0.63	0.67	4.64	2.99	5.35	5.13	6.21		4.37	10.30

### Genotypic classification based on tolerance to Orobanche

**Fig. 2:**
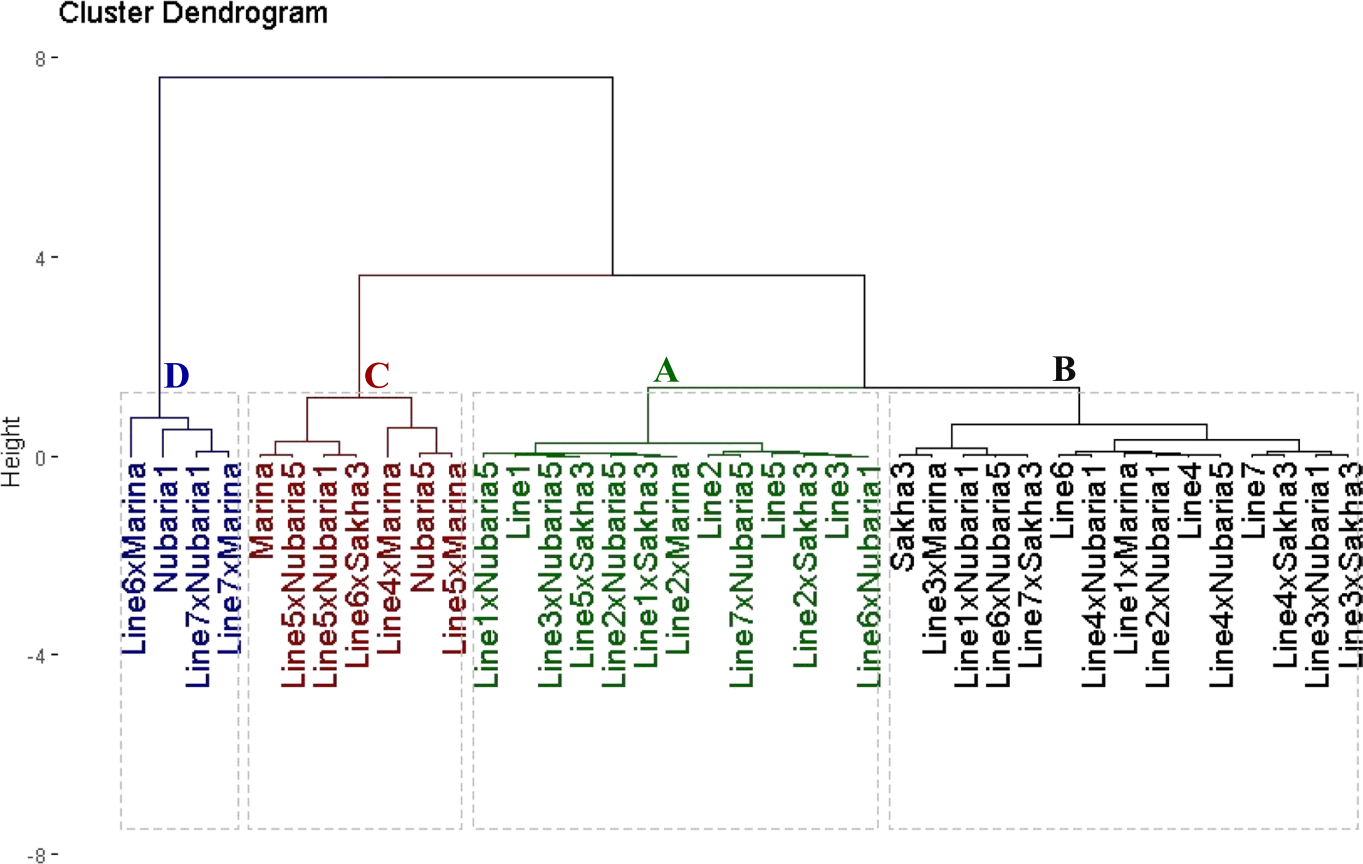
Dendrogram illustrating the phenotypic distances among faba bean lines, testers and their crosses based on stress susceptibility index

The stress susceptibility index determined based on seed yield under *Orobanche*-free and infested soils was employed to categorize the assessed lines, testers, and their crosses into different groups. The evaluated genotypes were grouped into four clusters utilizing cluster analysis (Fig. 2). Group A comprised 13 genotypes (four lines and nine crosses) exhibiting the lowest stress susceptibility values indicating high tolerance to *Orobanche*. Group B encompassed 15 genotypes (3 lines, one tester, and 11 crosses) with intermediate-low stress susceptibility values, categorized as moderately tolerant to *Orobanche.* Group C included 7 genotypes (2 testers and 5 crosses) recording intermediate-high stress susceptibility values, classified as moderately susceptible to *Orobanche*. Group D consisted of 4 genotypes (one tester and three crosses) demonstrating the highest stress susceptibility values, identified as highly susceptible to *Orobanche.*

### Interrelationships among assessed genotypes and studied traits under Orobanche-infested soil

**Fig. 3:**
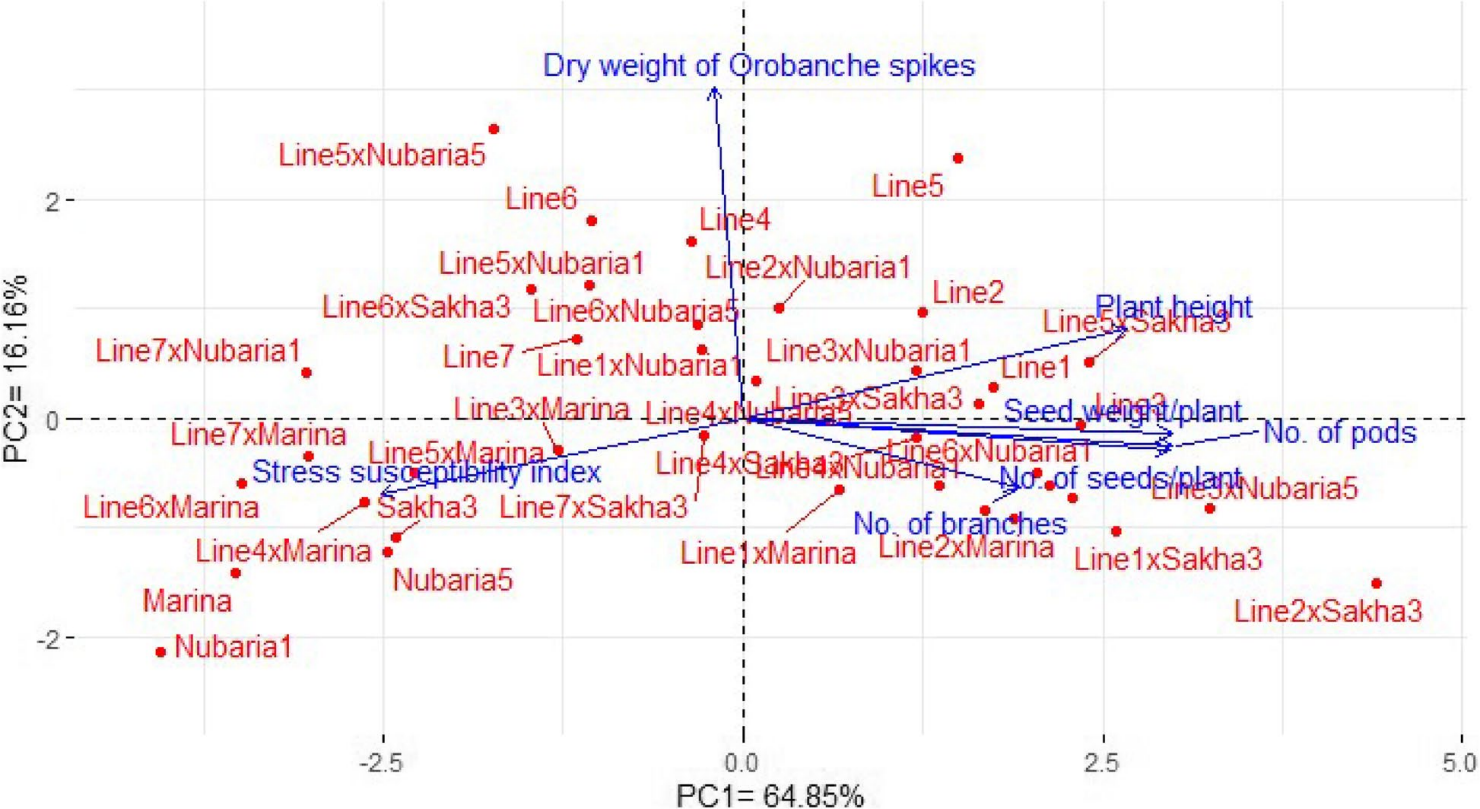
PCA biplot for the evaluated faba bean lines, testers, and their crosses based on the evaluated agronomic traits under *Orobanche*-infested soil

Principal component analysis was employed to explore the association among the assessed faba bean genotypes and studied agronomic traits under *Orobanche*-infested soil (Fig. 3). The first two PCs accounted for 81.01% of the variability, hence were utilized to perform the biplot (Table 4). PC1 explained 64.85% of the total variation and primarily illustrated agronomic performance of the evaluated faba bean lines, testers, and their crosses. The genotypes were dissimilar with diverse multidimensional spaces and different distance plots. The PC1 divided the genotypes on both sides based on their agronomic performance and stress susceptibility index. The high-yielding faba bean genotypes with lowest stress susceptibility index were positioned on the positive side as Line1×Nubaria5, Line5×Sakha3, Line2×Sakha3, Line3×Nubaria5, Line7×Nubaria5, Line6×Nubaria1, Line5, Line1×Sakha3, Line3, Line3×Sakha3, and Line2×Nubaria5. The aforementioned genotypes exhibited a positive association with seed yield and its related traits and a negative association with stress susceptibility index. Conversely, the lowest-performing genotypes with high-stress susceptibility index were situated on the extremely negative side as Line7×Nubaria1, Line7×Marina, Line6×Marina, Line4×Marina, Marina, and Nubaria1. These genotypes exhibited a negative association with seed yield and its related traits and a positive association with stress susceptibility index. The seed yield and its related traits exhibited significant interrelationships with a negative association with stress susceptibility index, and weight of *Orobanche* spikes.

**Table 4:** Loading values of Principal Component Analysis (PCA) for the five main components (PC1-PC5) for evaluated agronomic traits of assessed faba bean genotypes under *Orobanche*-infested soil conditions

Variable	PC1	PC2	PC3	PC4	PC5
Plant height	0.859	0.262	0.260	0.026	-0.352
Number of branches	0.619	-0.205	0.734	-0.112	0.154
Number of pods	0.962	-0.082	-0.131	0.159	0.039
Number of seeds per plant	0.954	-0.095	-0.195	0.180	0.036
Seed weight per plant	0.958	-0.047	-0.127	0.102	0.124
Dry weight of *Orobanche* spikes	-0.066	0.976	0.115	0.109	0.133
Stress susceptibility index	-0.812	-0.223	0.291	0.454	-0.030
Eigenvalue	4.54	1.13	0.77	0.30	0.18
Variance percent (%)	64.85	16.16	11.07	4.27	2.63
Cumulative (%)	64.85	81.01	92.08	96.35	98.98

### General combining ability effects

The general combining ability (GCA) effects of lines and testers for the studied traits are presented in Table 5. Desirable significant and positive GCA for plant height were obtained by Line5, Line7, Nubaria5, and Marina under *Orobanche* free soil and Line1, Line2, and Line4, and the testers Nubaria1, Sakha3, and Nubaria5 under *Orobanche* infested soil. The tester Nubaria1 displayed desirable positive and significant GCA for number of branches/plant under *Orobanche*-free soil. Line2, Line3, and Line4, in addition to Nubaria1 and Sakha3, showed desirable positive and significant GCA for the number of branches/plant under Orobanche-infested soil. Line1 and Marina revealed desirable positive GCA for the number of pods/plant under Orobanche-free soil. Moreover, Line1, Line2, Line3, Sakha3, and Nubaria5 revealed desirable positive GCA for the number of pods/plant under *Orobanche*-infested soil. Line1 and Marina revealed desirable positive GCA for number of seeds/plant under *Orobanche*-free soil. Line1, Line2, Line3, Sakha3, and Nubaria5 displayed significantly desirable GCA for the number of seeds/plant under Orobanche-infested soil. Line1, Line3, and Line7, as well as Nubaria1 and Sakha3, exhibited significant desirable GCA for seed weight/plant under *Orobanche*-free soil. Line1, Line2, Line3, Sakha3, and Nubaria5 gave significantly desirable GCA for seed weight/plant under *Orobanche*-infested soil. Line1, Line2, and Line4, as well as the tester Sakha3 and Marina, showed significantly desirable negative GCA for the dry weight of *Orobanche* spikes/plot (g).

**Table 5:** General combing ability for the studied traits under both *Orobanche*-free and infested soils conditions

**Genotypes**	**Plant height**		**No. of branches**		**No. of pods/plant**	
	**Free**	**Infested**	**Free**	**Infested**	**Free**	**Infested**
**Lines**						
Line1	0.43	11.08 **	0.27	0.03	13.26 **	2.85 **
Line2	-0.2	8.55 **	-0.02	0.49 **	-0.09	6.92 **
Line3	-2.01 **	-0.77	-0.14	0.39 *	1.31	3.48 **
Line4	-1.72 **	4.12 **	-0.04	0.37 *	-0.01	-2.14 **
Line5	1.08 **	-7.6 **	0.27	-0.46 **	-3.49 **	-2.92 **
Line6	0.21	-7.02 **	-0.11	-0.75 **	-4.83 **	-3.31 **
Line7	2.22 **	-8.35 **	-0.23	-0.08	-6.15 **	-4.89 **
LSD0.05 (gi-gj)	0.8	1.01	0.31	0.34	2.32	1.5
**Testers**						
Nubaria1	-2.23 **	2.42 **	0.6**	0.34 *	-2.1 *	-1.81*
Sakha3	-2.72 **	8.29 **	0.23	0.34 *	-0.42	3.28**
Nubaria5	0.99 *	5.66 **	-0.06	0.15	-0.85	2.12*
Marina	3.96**	-16.37 **	-0.77**	-0.83 **	3.37 *	-3.59**
LSD0.05 (gi-gj)	0.6	0.76	0.24	0.25		
**Genotypes**	**No. of seeds/plant**		**Seed weight/plant**		**No. of *Orobanche *spikes**	**Dry weight of *Orobanche *spikes/plot (g)**
	**Free**	**Infested**	**Free**	**Infested**	**Infested**	**Infested**
**Lines**						
Line1	24.01 **	11.07 **	3.46 *	6.14 **	3.64 *	-57.14 **
Line2	-4.21 **	20.57 **	-7.65 **	11.57 **	-25.68 **	-38.59 **
Line3	-0.74	6.63 **	3.3 *	8.07 **	12.17 **	0.54
Line4	-2.82 *	-7.07 **	-11.1 **	-7.35 **	4.27 *	-31.81 **
Line5	-3.68 **	-7.88 **	2.37	-6.79 **	22.93 **	108.66 **
Line6	-6.98 **	-9.63 **	-2.3	-5.2 **	-8.45 **	20.52 **
Line7	-5.57 **	-13.68 ^**^	11.93 **	-6.44 **	-8.88 **	-2.19
LSD0.05 (gi-gj)	2.68	2.57	3.11	2.19	4.14	5.15
**Testers**						
Nubaria1	-8.11 **	-7.25**	3.24*	-2.29*	12.79 **	48.33 **
Sakha3	1.21	11.97**	3.84*	9.62**	0.47	-10.73 **
Nubaria5	-2.06	7.54**	-2.98*	6.02**	3.67 *	13.79 **
Marina	8.96 **	-12.26**	-4.1*	-13.36**	-16.94 **	-51.38 **
LSD0.05 (gi-gj)			2.35	1.65	3.13	3.89

* and ** signify *P* value < 0.05 and 0.01, in the same order

### Specific combining ability

Specific combining ability (SCA) effects of 28 studied crosses for the examined traits under the two types of soils are presented in Table 6. Desirable positive and significant SCA for plant height were detected by the crosses Line1×Marina, Line2×Marina, Line3×Nubaria5, Line4×Nubaria5, Line4×Sakha3, Line5×Nubaria1, and Line6×Marina under *Orobanche* free soil, in addition to the crosses Line1×Marina, Line2×Sakha3, Line2×Marina, Line3×Nubaria1, Line3×Nubaria5, Line4×Nubaria1, Line4×Sakha3, Line5×Nubaria1, Line5×Sakha3, Line6×Nubaria1, Line6×Nubaria5, Line7×Nubaria5 and Line7×Marina under *Orobanche* infested soil. The crosses Line1×Nubaria5, Line5×Marina, and Line6×Sakha3 displayed desirable positive and significant SCA for number of branches/plant under *Orobanche*-free soil. The crosses Line1×Marina, Line2×Sakha3, Line4×Nubaria1, and Line7×Marina showed desirable positive and significant SCA for number of branches/plant under *Orobanche*-infested soil. The crosses Line1×Marina, Line2×Nubaria1, Line3×Nubaria5, Line4×Sakha3, Line5×Marina, and Line7×Marina had desirable positive SCA for number of pods/plant under *Orobanche* free soil. The crosses Line1×Marina, Line2×Sakha3, Line4×Marina, Line3×Nubaria5, Line4×Nubaria1, Line5×Sakha3, Line6×Nubaria1, and Line7×Nubaria5 revealed desirable positive SCA for number of pods/plant under *Orobanche* infested soil. The crosses Line1×Nubaria5, Line1×Marina, Line2×Sakha3, Line3×Nubaria1, Line3×Nubaria5, Line4×Nubaria1, Line6×Sakha3, Line7×Nubaria1 and Line7×Marina showed SCA for number of seeds/plant under *Orobanche* free soil. The crosses Line1×Sakha3, Line2×Sakha3, Line2×Marina, Line3×Nubaria5, Line4×Nubaria1, Line5×Sakha3, Line5×Marina, Line6×Nubaria1 and Line7×Nubaria5 gave significant desirable SCA for number of seeds/plant under *Orobanche* infested soil. The crosses Line1×Nubaria5, Line3×Nubaria5, Line5×Marina, Line6×Marina, and Line7×Nubaria1 exhibited significant desirable SCA for seed weight/plant under *Orobanche* free soil. The crosses Line1×Nubaria5, Line1×Marina, Line2×Sakha3, Line2×Marina, Line3×Nubaria5, Line4×Nubaria1, Line5×Sakha3, Line6×Nubaria1 and Line7×Nubaria5 gave significant desirable SCA for seed weight/plant under *Orobanche* infested soil. The crosses Line1×Sakha3, Line1×Nubaria5, Line2×Sakha3, Line2×Nubaria5, Line3×Nubaria5, Line4×Nubaria1, Line5×Nubaria1, Line5×Sakha3, Line5×Marina, Line6×Nubaria1, Line6×Marina, Line7×Sakha3 and Line7×Nubaria5 showed significant desirable negative SCA for weight of *Orobanche* spikes under *Orobanche* infested soil.

### Heterosis

Heterosis expressed as the increment percentage of the F_1_ crosses above the mid-parent is listed in Table 7. All crosses obtained desirable positive and significant mid-parent heterosis for plant height under all conditions except for Line4×Marina and Line5×Marina under *Orobanche*-infested soil. Desirable positive and significant mid-parent heterosis estimates for the number of branches of/plant were detected in fourteen crosses under *Orobanche*-free soil and nine crosses under *Orobanche*-infested soil. Only three crosses had undesirable negative mid-parent heterosis under *Orobanche*-infested soil. Twenty-one and eight crosses gave desirable positive and significant mid-parent heterosis for number of pods/plant under *Orobanche*-free and infested soils, respectively. Only eight crosses had undesirable negative mid-parent heterosis under *Orobanche*-infested soil. Also, there was a desirable positive significant mid-parent heterosis for number of seeds/plant by twenty-six and ten crosses under *Orobanche*-free and infested soils, respectively. Only eleven crosses had undesirable negative mid-parent heterosis under *Orobanche*-infested soil. Preferable positive and significant mid-parent heterosis for seed weight/plant was obtained by twenty-six and fifteen crosses under *Orobanche*-free and infested soils, respectively. Only eleven ten crosses had undesirable negative mid-parent heterosis under *Orobanche*-infested soil. Fifteen crosses detected preferable negative and significant mid-parent heterosis for the weight of *Orobanche* spikes.

**Table 6:** Specific combing ability for the studied traits under both *Orobanche*-free and infested soils conditions

Crosses	Plant height		No. of branches		No. of pods per plant		No. of seeds per plant		Seed weight per plant		Dry weight of Orobanche spikes/plot (g)
	*O*.- Free	*O.* Infested	*O*.- Free	*O*.- Infested	*O*.- Free	*O*.- Infested	*O*.- Free	*O*.- Infested	*O*.- Free	*O*.- Infested	*O*.- Infested
Line1×Nubaria1	-2.08**	-13.94**	-0.35	-0.69*	-4.24*	-3.36*	-10.86**	-12.49**	-5.24*	-10.08**	63.6 **
Line1×Sakha3	-3.26**	-1.09	-0.16	-0.27	-3.12	1.01	-4.48*	5.56*	-3.72	2.42	-49.38 **
Line1×Nubaria5	0.94	-2.18*	0.71**	0.42	2.56	-0.02	10.42**	3.04	7.27**	3.68*	-29.07 **
Line1×Marina	4.4**	17.2**	-0.2	0.54*	4.8*	2.38*	4.91*	3.89	1.69	3.98*	14.85 **
Line2×Nubaria1	-3.95**	-14.9**	0.29	-0.51	8.89**	-5.28**	-1.72	-16.67**	0.7	-12.69**	111.93 **
Line2×Sakha3	-0.14	5.71**	0.28	0.83**	0.33	3.55**	8.41**	11.54**	4.57	10.41**	-70.61 **
Line2×Nubaria5	-0.09	-4.31**	0.3	-0.33	-5.94**	-1.33	-1.96	-6.52**	0.07	-7.84**	-33.61 **
Line2×Marina	4.18**	13.51**	-0.86**	0.01	-3.29	3.06*	-4.74*	11.65**	-5.35*	10.13**	-7.70
Line3×Nubaria1	-2.08**	7.84**	-0.48	0.31	-5.21*	-0.28	9.88**	-6.02**	-1.60	2.71	7.46
Line3×Sakha3	-3.08**	-3.95**	0.10	-0.1	-0.22	-1.56	-4.73*	-9.72**	-0.39	-6.77**	42.69 **
Line3×Nubaria5	5.26**	4.45**	0.06	0.17	6.57**	5.77**	7.8**	22.27**	12.33**	10.19**	-71.32 **
Line3×Marina	-0.1	-8.34**	0.32	-0.38	-1.14	-3.93**	-12.96**	-6.54**	-10.35**	-6.13**	21.17 **
Line4×Nubaria1	-0.06	7.79**	0.38	1.43**	0.97	3.95**	4.25*	11.74**	2.73	8.08**	-55.69 **
Line4×Sakha3	2.53**	4.41**	-0.38	-0.24	5.48**	-0.14	1.75	-0.49	1.96	-0.22	11.15 *
Line4×Nubaria5	1.77*	1.04	-0.11	-0.88**	1.98	-1.68	-4.07	-5.53*	-6.32*	-6.25**	23.69 **
Line4×Marina	-4.24**	-13.23**	0.11	-0.31	-8.43**	-2.13	-1.93	-5.72*	1.64	-1.62	20.85 **
Line5×Nubaria1	5.31**	9.75**	0.14	-0.28	-1.45	-1.47	-0.18	-3.83	-0.16	-3.96*	-45.44 **
Line5×Sakha3	1.19	13.55**	-0.75**	0.42	-5.13*	5.11**	-0.88	21.03**	-0.27	18.03**	-36.50 **
Line5×Nubaria5	-3.8**	-11.85**	-0.45	0	1.63	-5.59**	-3.09	-21.98**	-11.59**	-16.9**	176.6 **
Line5×Marina	-2.7 **	-11.44 **	1.07 **	-0.14	4.95 *	1.95	4.15	4.79 *	12.02 **	2.82	-94.62 **
Line6×Nubaria1	-1.83 **	14.68 **	-0.21	0.35	0.76	8.95 **	-8.7 **	35.42 **	-12.85 ^**^	29.42 **	-129.87 **
Line6×Sakha3	1.25	-18.25 **	0.63 *	-0.27	2.24	-5.4 **	4.34 *	-19.56 ^**^	4.20	-16.98 ^**^	118.98 **
Line6×Nubaria5	-1.37 *	10.6 **	-0.44	0.2	-0.73	-2.56 *	3.13	-10.62 ^**^	3.30	-7.59 **	27.36 **
Line6×Marina	1.95 **	-7.02 **	0.01	-0.28	-2.27	-0.99	1.22	-5.24 *	5.35 *	-4.85 *	-16.47 **
Line7×Nubaria1	4.69 **	-11.21 **	0.24	-0.6 *	0.27	-2.51 *	7.32 **	-8.15 **	16.42 **	-13.49 ^**^	48.01 **
Line7×Sakha3	1.49 *	-0.37	0.28	-0.37	0.42	-2.58 *	-4.41 *	-8.36 **	-6.35 *	-6.89 **	-16.32 **
Line7×Nubaria5	-2.7 **	2.25 *	-0.07	0.43	-6.07 **	5.41 **	-12.25 ^**^	19.34 **	-5.07 *	24.7 **	-93.59 **
Line7×Marina	-3.48 **	9.34 **	-0.45	0.55 *	5.38 **	-0.33	9.34 **	-2.83	-5.00 *	-4.32 *	61.91 **
LSD0.05 (Sij-Sik)	1.60	2.02	0.63	0.67	4.64	2.99	5.35	5.13	6.21	4.37	10.30

* and ** signify *P* value < 0.05 and 0.01, in the same order

**Table 7:** Heterosis above the mid-parent for the studied traits under both *Orobanche*-free and infested soil conditions

Cross	Plant height		No. of branches		No. of pods per plant		No. of seeds per plant		Seed weight per plant		Dry weight of *Orobanche *spikes/plot (g)
	Free	Infested	Free	Infested	Free	Infested	Free	Infested	Free	Infested	Infested
Line1×Nubaria1	3.05**	21.7**	21.43	-2.75	48.43*	7.09**	30**	-4.88**	21.98**	8**	84.03**
Line1×Sakha3	6.33**	46.63**	19.54	46.9	39.7**	75.01	37.7**	68.01**	23.35**	65.23**	-45.72
Line1×Nubaria5	5.9**	30.14**	58.73**	18.35	54.38**	70.64	39.17**	75.68	35.06**	88.96**	-22.54**
Line1×Marina	8.24**	40.08**	60.00*	30.08	89.52**	40.61	93.4**	28.09**	86.72**	23.16**	-20.59**
Line2×Nubaria1	3.23**	26.03**	33.56	35.57	94.11**	23.42**	34.86**	13.66**	23.51**	24.32**	85.49**
Line2×Sakha3	11.09**	63.15**	27.73	158.37**	45.34**	124.17**	47.66**	108.92**	28.02**	115.85**	-57.43
Line2×Nubaria5	6.84**	33.3**	49.12**	25.57	24.87**	88.61	24.09**	77.3	19.59**	68.34	-29.15**
Line2×Marina	9.87**	42.25**	38.63	54.6*	68.33**	79.19	89.27**	84.45	69.05**	81.55	-39.59**
Line3×Nubaria1	7.02**	42.92**	26.32	46.5**	39*	11.44**	27.66*	-16.16**	27.7**	51.86**	96**
Line3×Sakha3	10.87**	37.48**	33.11	79.93**	41.64**	24.84**	16.88	1.44**	28.65**	26.14*	62.39**
Line3×Nubaria5	13.71**	32.15**	56.69**	26.85	63.03**	81.06	17.62**	83.74	43.01**	96.94**	-8.81**
Line3×Marina	8.5**	0.18**	104.4**	13.51	72.57**	-34.99**	47.81	-32.66**	66.58**	-25.55**	35.63**
Line4×Nubaria1	5.22**	42.79**	27.4*	91.54**	53.86**	100.79	31.5**	113.73**	27.6**	157.76**	-19.32**
Line4×Sakha3	12.07**	48.18**	7.23	83.83**	53.08**	70.03*	31.28**	67.02**	25.96*	76.84**	-20.33**
Line4×Nubaria5	7.08**	28.58**	28.74**	-2.13	42.42**	55.57	14.57**	67.14	11.54	69.71*	-8.35**
Line4×Marina	1.69**	-4.22**	55.6	23.26	39.63*	-31.46**	75.75**	-26.22**	90.64**	-27.67**	-29.07**
Line5×Nubaria1	14.7**	32.26**	38.22*	21.62	34.93	-33.42**	42.18**	-41.27**	31.65**	-37.77**	28.13**
Line5×Sakha3	15.99**	46.7**	14	119.8**	11.19	60.87	43.27**	53.22	30.93**	47.48	2.25**
Line5×Nubaria5	6.99**	1.34**	39.82**	15.16	32.13**	-39.95**	27.46**	-55.93**	13.84*	-57.99**	86.14**
Line5×Marina	7.43**	-15.83**	119.49**	15.43	76.71**	-20.64**	111.83**	-32.08**	116.22**	-52.89**	-31.82**
Line6×Nubaria1	8.51**	59.71**	22.74	14.24	28.4	252.1**	18.53*	324.57**	11.59	310.59**	-37.54**
Line6×Sakha3	16.2**	22.94**	34.3	27.53	21.93	11.24	34.6**	-6.26	33.11**	-11.24	40.75**
Line6×Nubaria5	9.18**	45.54**	29.77**	-4.29	13.1	63.38	21.4**	53.58	29.93**	57.02	5.99**
Line6×Marina	11.43**	4.38**	68.12**	-28.57**	36.09	-11.11	83.02**	-24.02**	99.32**	-45.67**	-29.57**
Line7×Nubaria1	16.25**	20.71**	32.62	15.79	54.85**	-32.73**	64.56**	-29.97**	77.89**	-38.04**	92.19**
Line7×Sakha3	18.49**	47.49**	27.4	77.37**	38.25**	25.85	51.18**	29.74*	45.8**	32.93*	2.92**
Line7×Nubaria5	9.93**	32.96**	39.96**	36.58	14.86	170.03**	27.11*	223.54**	46.3**	263.7**	-34.31
Line7×Marina	8.72**	26.91**	53.88*	61.36*	98.52**	-32.05*	147.03**	-32.54**	127.18**	-51.85**	40.95**

* and ** signify *P* value < 0.05 and 0.01, in the same order

### Biplot analysis for general combining ability

**Fig. 4:**
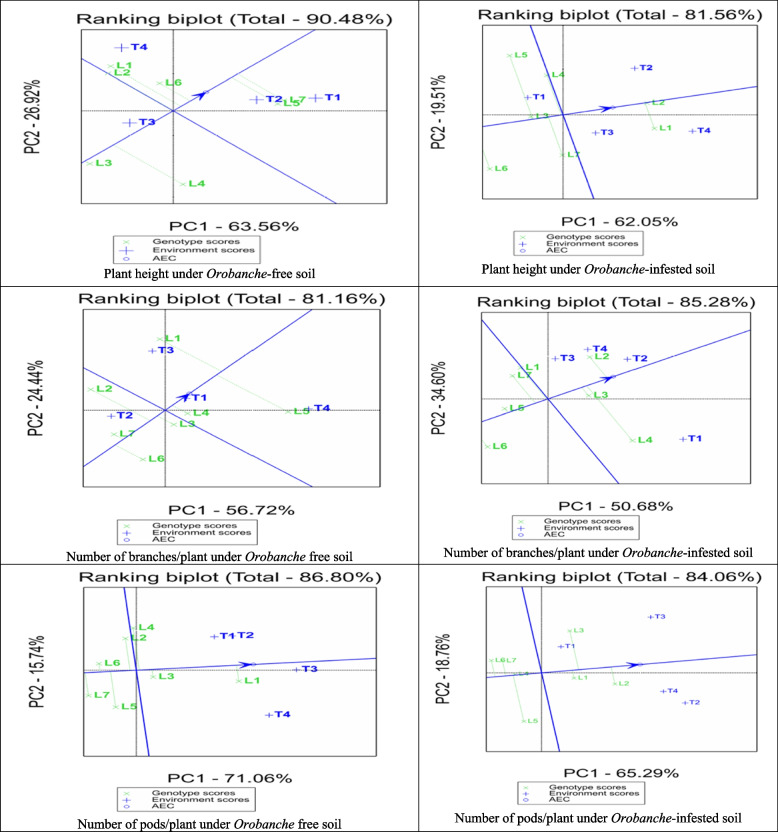
GGE biplot for number of branches per plant, plant height, and number of pods per plant under *Orobanche*-free and infested soil conditions showing average tester coordinate view of four testers and lines parents. Parents showed that blue and green colors are testers and lines, respectively

The Average Tester Coordination (ATC) view of the biplot brought up by GGE biplot for the studied traits under *Orobanche*-free and infested soils (Fig. 4). The GGE biplots explain most of the variation in the studied traits, ranging from 68.44 of the total variation for number of *Orobanche* spikes to 90.48 for plant height under *Orobanche*-free soils of the total variation. Plant height of the assessed lines under *Orobanche*-free soil was ranked as Line7 > Line5 > Line6 > Line1 ≈ Line2, and only Line3 and Line4 had negative GCA effects. Similarly, the testers were ranked as Nubaria1 > Sakha3 > Marina > Nubaria, and only Nubaria5 had negative GCA effects. While under *Orobanche*-infested soil, the lines were ranked as Line2 > Line1 > Line4 > Line7 > Line5 ≈ Line3 > Line6, and the testers were in the order Marina > Sakha3 > Nubaria5 > Nubaria1. Positive GCA effects were obtained only by Line2, Line1, Line4, Marin, Sakha3, and Nubaria5.

Number of branches/plant of assessed lines under *Orobanche* free soil were ranked as Line1 > Line5 > Line4 > Line3 > Line7 > Line6, and the testers were ranked as Nubaria5 > Marina > Nubaria1 > Sakha3. Positive GCA effects were obtained only by Line1, Line5, Line4, Nubaria5, Marina, and Nubaria1. While under *Orobanche*-infested soil, the lines were ranked as Line2 > Line4 ≈, Line3 > Line1 > Line7 > Line5 > Line6, and the testers were in the order Sakha3 > Marina ≈ Nubaria1 > Nubaria5. Only Line2, Line4, Line3, and Line1 and all testers obtained positive GCA effects. Number of pods/plant of assessed lines under *Orobanche* free soil were ranked as Line1 > Line3 > Line2 ≈, Line4 > Line5 > Line6 > Line7and the testers were ranked as Nubaria5 > Marina ≈ Sakha3 > Nubaria1. Only Line1, Line3, Line2, Line4, and all testers obtained positive GCA effects. While under *Orobanche*-infested soil, the lines were ranked as Line2 > Line3 > Line1 > Line4 ≈ Line5 > Line7 > Line6, and the testers were in the order Sakha3 ≈ Nubaria5 ≈ Marina > Nubaria1. Only Line2, Line3, Line1, and all testers obtained positive GCA effects.

**Fig. 5:**
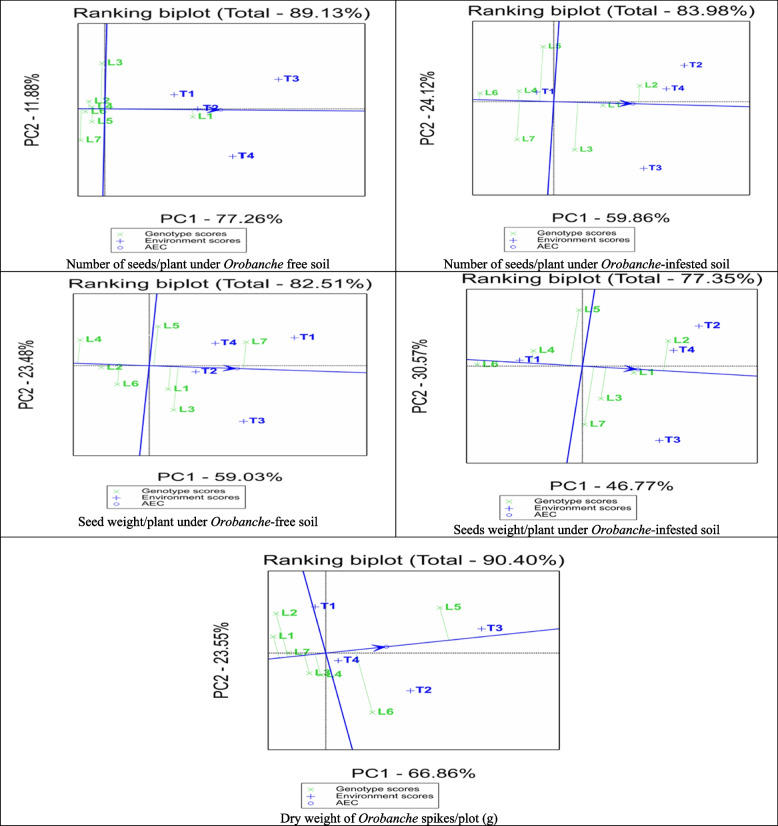
GGE biplot for number of seeds/plant, seed weight per plant, and weight of Orobanche spikes under Orobanche -free and infested soil conditions showing average tester coordinate view of four testers and lines parents. Parents showed that blue and green colors are testers and lines, respectively

Number of seeds/plant of assessed lines under *Orobanche* free soil were ranked as Line1 > Line3 > Line4 ≈ Line5 ≈ Line6 ≈ Line2 > Line7and the testers were ranked as Nubaria5 > Marina > Sakha3 > Nubaria1 (Fig. 5). Positive GCA effects were obtained only by Line1 and all testers. While under *Orobanche*-infested soil the lines were ranked as Line2 > Line1 > Line3 > Line5 > Line4 ≈ Line7 > Line6, and the testers were in the order Sakha3 > Marina > Nubaria5 > Nubaria1. Positive GCA effects were obtained only by Line2, Line1, Line3, and all testers, except for Nubaria1. Seed weight/plant of assessed lines under *Orobanche*-free soil were ranked as Line7 > Line3 > Line1 > Line5 > Line6 > Line2 > Line4, and the testers were in the order of Nubaria1 > Nubaria5 > Marina > Sakha3. Positive GCA effects were obtained by Line7, Line3, Line1, Line5, and all testers. While under *Orobanche*-infested soil, the lines were ranked as Line2 > Line1 > Line3 > Line7 > Line5 > Line4 > Line6, and the testers were ranked as Sakha3 > Marina > Nubaria5 > Nubaria1. All parents obtained positive GCA effects except for Line6, Line4, Line5, and Nubaria1.

Positive GCA effects were obtained only by all parents except for Line2, Line7, Line1, Line4 and Marin.Likewise, weight of *Orobanche* spikes of assesed lines were ranked as Line5 > Line6 > Line4 > Line3 > Line7 > Line2 > Line1, and the testers were ranked as Nubaria5 > Sakha3 > Marina > Nubaria1. Only Line5, Line6, Line4, and all testers obtained positive GCA effects.

### Best crosses between lines and testers

**Fig. 6:**
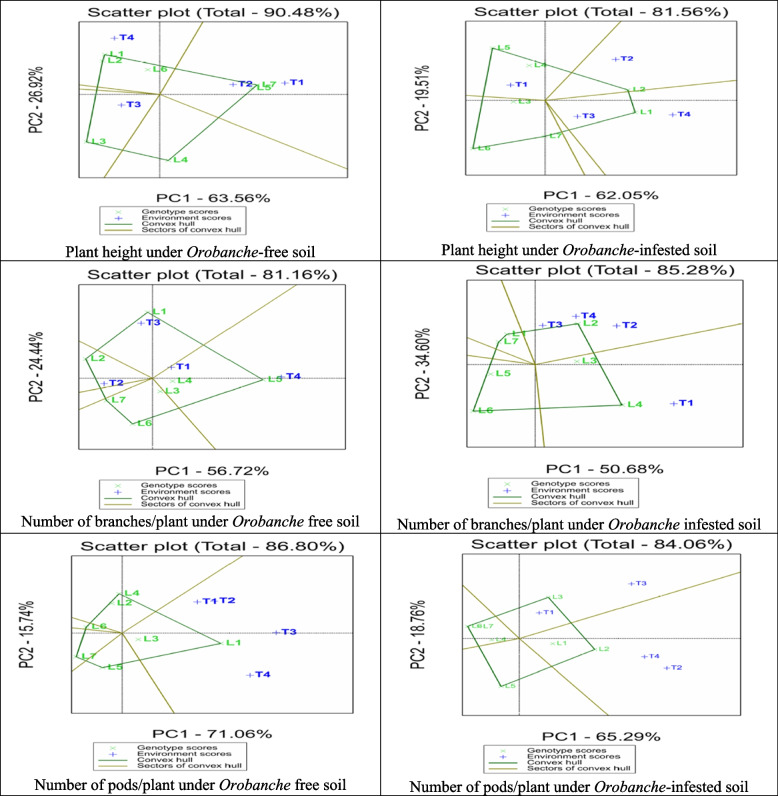
GGE biplot, for number of branches per plant, plant height, and number of pods per plant under *Orobanche*-free and infested soil conditions showing polygon view of four testers and seven lines parents. Parents showed that blue and green colors are testers and lines, respectively

The polygon view of the biplot for the studied characters under *Orobanche*-free and infested soils is displayed in Fig. 6. The biplot of plant height under *Orobanche*-free soil was divided into four sectors with Line4, Line7, Line1, and Line3 as the vertex entries and are described as 4, 7, 1, and 3, in the same order. No tester fell in the Line4 sector, indicating that Line4 was not the best mating partner with any of the four testers. The highest SCAs were related to Line5 and Line7, with the two testers, Nubaria1 and Sakha3, included in the sector of Line7. In the sector of Line1, SCAs were related to Line1 and Line2 with Marine. The highest SCAs were related to Line3, included in that sector, with the Nubaria5. On the other hand, plant height under *Orobanche*-infested soil was divided into four sectors, with Line1, Line2, Line5, and Line6 as the vertex entries. No tester fell in the Line6 sector, suggesting that Line6, Line7, and Line3 were not the best mating partners with any of the four testers. The highest SCAs were related to Line2 with Sakha3, Line1 with the two testers Marina and Nubaria5, and Line4 and Line5 with the tester Nubaria1.

The biplot for number of branches/plant under *Orobanche*-free soil was divided into five sectors, with Line5, Line1, Line2, Line7, and Line6 as the vertex entries. No tester fell in Line6 and Line7 sectors, suggesting that Line6, Line7, Line4, and Line3 were not the best mating partners with any of the four testers. The highest SCAs were related to Line5 with Nubaria1 and Marine, Line1 with the tester Nubaria5, and Line2 with the tester Sakha3. The biplot for number of branches/plant under *Orobanche*-infested soils was divided into five sectors, with Line4, Line2, Line1, Line7, and Line6 as the vertex entries. No tester fell in Line1, Line6, and Line7 sectors, suggesting that Line6, Line7, and Line1 were not the best mating partners with any of the four testers. The highest SCAs were related to Line4 with Nubaria1 and Line2 with the remaining three testers.

The biplot for number of pods/plant under *Orobanche*-free soils was divided into five sectors, with Line1, Line4, Line6, Line7, and Line5 as the vertex entries. Only Line1 had the highest SCAs with the four testers, while other sectors were without any testers, suggesting that all lines except Line1 were not the best mating partner with any of the four testers. The biplot for number of pods/plant under *Orobanche*-infested soils was divided into four sectors, with Line2, Line3, Line6, and Line5 as the vertex entries. No tester fell in Line6 and Line5 sectors, suggesting that Line6, Line7, and Line1 were not the best mating partners with any of the four testers. The highest SCAs were related to Line2 with Sakha3 and Marina and Line3 with the two testers Nubaria1 and Nubaria5.

**Fig. 7:**
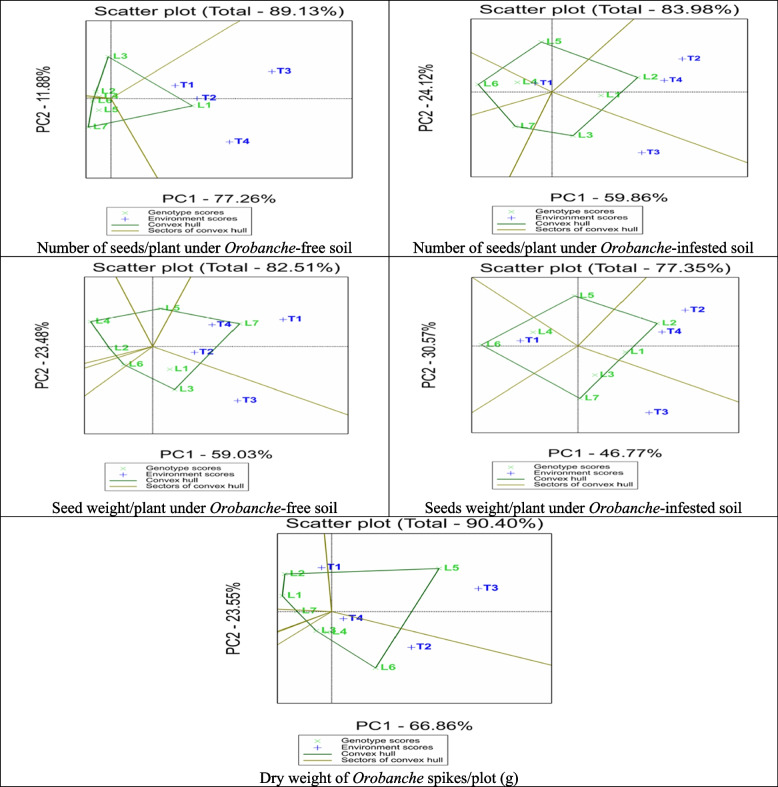
GGE biplot, for number of seeds/plant, seed weight per plant, and weight of *Orobanche* spikes under *Orobanche*-free and infested soil conditions showing polygon view of four testers and seven lines parents. Parents showed that blue and green colors are testers and lines, respectively

The biplot for number of seeds/plant under *Orobanche*-free soils was separated into three sectors, with lines 1, 3, and 7 as the vertex entries (Fig. 7). Only Line1 had the highest SCAs with the four testers, while the two other sectors were without any testers, suggesting that all lines except Line1 were not the best mating partner with any of the four testers. The biplot for the number of seeds/plant under *Orobanche*-infested soils was divided into five sectors, with Line2, Line5, Line6, Line7, and Line3 as the vertex entries. No tester fell in Line6 and Line7 sectors, suggesting that Line1, Line4, Line6, and Line7 were not the best mating partners with any of the four testers. The highest SCAs were related to Line2 with the two testers Sakha3 and Marin, Line3 with the tester Nubaria5, and Line5 with the tester Nubaria1.

The biplot for seed weight/plant under *Orobanche*-free soils was divided into five sectors, with Line7, Line5, Line4, Line6, and Line3 as the vertex entries. Only Line3 with the tester Nubaria5 and Line7 with the other three testers had the highest SCAs, while the other sectors were without any tester, suggesting that all lines except Line3 and Line7 were not the best mating partner with any of the four testers. The biplot for seed weight/plant under *Orobanche*-infested soils was divided into four sectors, with Line2, Line5, Line6, and Line7 as the vertex entries. No tester fell in the Line5 sector, indicating that Line1, Line3, Line4, and Line5 were not the best mating partners with any of the four testers. The highest SCAs were related to Line2 with the two testers Sakha3 and Marin, Line7 with the tester Nubaria5, and Line6 with the tester Nubaria1.

The highest SCAs were related to Line3 with the tester Sakha3, Line5 with the two testers Nubaria1 and Nubaria5, and Line1 with the tester Marin. The biplot for the dry weight of the *Orobanche* spike/plot (g) was divided into five sectors, with Line5, Line2, Line1, Line3, and Line6 as the vertex entries. No tester fell in the Line5 sector, suggesting that Line1, Line3, Line4, and Line5 were not the best mating partners with any of the four testers. The highest SCAs were related to Line5 with the tester Nubaria5, Line1 and Line2 with the tester Nubaria1, and Line3 and Line6 with the two testers Sakha3 and Marina.

### Genetic variance and contribution to the total variance

The gene action and the contribution of faba bean lines, testers, and their crosses in the studied traits are shown in Table 8. The additive variance was negative and estimated to be zero for number of *Orobanche* spikes. Dominance variances were higher than corresponding additive variances for all the studied traits under different conditions. Lines were more prominent and important for the number of pods/plant and seeds/plant under all conditions, and seed weight/plant under *Orobanche*-free and infested soils. For other traits, testers were more prominent and important. The line×tester interaction contributed predominantly for all traits under most conditions.

**Table 8:** Dominance, additive genetic components, and proportional contribution of lines, testers, and their crosses to total variance for the studied traits under both *Orobanche*-free and infested soil conditions

Trait		Dominance	Additive	Contribution of lines	Contribution of testers	Contribution of lines×testers
Plant height	**Free**	13.18	10.70	40.67	48.62	0.67
	**Infested**	157.97	22.18	37.27	40.55	13.61
Number of branches/plant	**Free**	0.24	7.06	53.48	39.47	0.03
	**Infested**	0.33	28.11	34.98	36.90	0.04
Number of pods/plant	**Free**	24.68	62.22	7.23	30.56	4.32
	**Infested**	19.22	44.21	20.92	34.88	2.47
Number of seeds/plant	**Free**	63.30	55.12	21.03	23.85	16.01
	**Infested**	270.58	45.98	11.45	42.57	21.14
Seed weight/plant	**Free**	68.76	21.32	11.43	67.25	5.56
	**Infested**	197.55	33.62	24.15	42.22	9.92
Dry weight of *Orobanche* spikes	**Infested**	7131.73	30.31	15.56	54.13	220.54

## Discussion

Faba bean production in Egypt faces multifaceted challenges related to agricultural, environmental, and economic factors across various geographical regions. Breeding high-yielding and tolerant faba bean genotypes to *Orobanche* is pivotal to sustain production and ensure global food security, particularly considering the challenges posed by population growth. For developing promising faba bean genotypes, novel re-combinations, and optimal combining abilities are crucial aspects [38]. Assessing agronomic performance per host plant in both *Orobanche*-free and infested soil provides deeper insights into the impact of parasitism on hosts [39]. In the present study, eleven parents and their 28 F_1_ crosses were evaluated under *Orobanche*-free and infested soils. The results demonstrated considerable variations among the evaluated genotypes, wide diversity among the parental materials, and heterotic effects for all studied agronomic traits under *Orobanche*-free and infested soil conditions. The significant differences and wide diversity within the parental materials and developed crosses highlighted the presence of substantial genetic variability and heterotic effects for further exploitation in genetic improvement of faba bean under *Orobanche*-free and infested soil conditions. Similarly, prior publications have demonstrated significant variations among faba bean genotypes in agronomic traits under *Orobanche*-free and infested soil conditions [40–42].

Seed yield displayed a reduction ranging from 30.9 to 71.3% in the tolerant lines while by 78.1 to 94.6% in the susceptible testers under *Orobanche* infestation. These findings reflect the important role of breeding tolerant faba bean genotypes to *Orobanche*, especially in infested fields. This coincides with previous studies of Trabelsi et al. [39], Soliman et al. [43]. Furthermore, Mohamed et al. [44] disclosed that the decrease in faba bean yield due to *Orobanche* infestation varied from 0.0 to 50%, depending on the level of infestation. Likewise, Ismail and Fakkar [45] elucidated that the presence of one and four *Orobanche* spikes reduced seed yield by 19.9% and 46.6% in the first season and by 14.3% and 50.0% in the second season, respectively.

Evaluating agronomic performance of parental lines and testers is an essential selection index. Line1, Line2, Line3, and Line5 were superior genotypes under *Orobanche* infestation exhibiting the highest values of studied agronomic traits. Similarly, Line3, Line7, and Line2 were the best in reducing the number of *Orobanche* spikes and dry weight. These findings concur with previous results of Soliman et al. [43]. Moreover, the developed crosses exhibited considerable variation in their agronomic performance. Line2×Sakha3, Line3×Nubaria5, Line7×Nubaria5, Line6×Nubaria1, Line5×Sakha3, Line1×Sakha3, and Line1×Nubaria5 were identified as the most successful crosses for seed yield and contributing d traits under *Orobanche* infestation, and the best in reducing the number of *Orobanche* spikes and dry weight. These crosses, based on these findings and stress susceptibility indices, could be considered promising sources of high seed yield and tolerance to *Orobanche crenata.*

Notably, highly *Orobanche*-susceptible genotypes exhibited low numbers and weights of *Orobanche* spikes, possibly due to early *Orobanche* spike emergence on susceptible plants compared to tolerant ones. These results could be explained by considering underground *Orobanche crenata* infection events [46]. Those non-emerged *Orobanche crenata* infection events were systematically considered by several authors to select tolerant faba bean [47]. The unreliability of considering solely the number and weight of *Orobanche* spikes was evident, emphasizing the risk of selecting falsely tolerant genotypes. Hence, relying on the stress susceptibility index and seed yield superiority was deemed more reliable for selecting high-yield and *Orobanche*-tolerant genotypes. According to Fischer and Maurer [34] stress sensitivity index serves as a measure of stress tolerance, quantifying yield loss reduction under *Orobanche*-infested soils compared to free soils. Thereby it characterizes the relative *Orobanche* tolerance among faba bean genotypes in effectively identifying tolerant genotypes [34]. The results indicated that the susceptibility index revealed that all faba bean genotypes were significantly affected by *Orobanche* infestation, resulting in a substantial reduction across all studied traits.

The breeding strategy for achieving high productivity in faba bean could involve the exploitation of heterosis through developing promising crosses [21]. The advantages of heterozygosity due to outcrossing in faba bean highlighting the effective heterosis resulting from combined allelic and interallelic genes is essential for improving and stabilizing faba bean productivity [23, 24, 28, 48, 49]. The results revealed both significant negative and positive heterosis across studied agronomic traits under *Orobanche*-free and infested soil conditions. While agronomic performance represents the realized value, the heterotic response serves as an estimate that emphasizes consideration of both when selecting cross combinations, particularly for commercial cultivation [24]. Detected significant negative and positive heterosis for traits such as number of branches per plant, plant height, number of pods per plant, number of seeds/plant^,^ and seed weight/plant are in consonance with the findings of Bishnoi et al. [21], Lal et al. [24], Soliman et al. [38], Abdalla et al. [48], Zeinab and Helal [49]. The substantial contribution of line×tester interaction over testers for the studied traits indicated higher specific combining ability variance estimates [21]. The evaluated lines were more prominent and important for number of pods/plant and number of seeds/plant under all conditions and seed weight/plant under *orobanche*-free and infested soils in addition to number of *Orobanche* spikes, indicating a predominant maternal influence which should be used in further breeding programs to allow crop improvement. For other traits, testers were more prominent and important. Superior faba bean parents with significant and positive GCA effects are important to be considered in breeding promising cultivars [48, 50]. Notably, high positive GCA values would be advantageous for several agronmic traits, while negative values would be beneficial in the selection process for the number and weight of *Orobanche* spikes under infestation. Line1, followed by Line2, Line3, Line5, and Line7, demonstrated desirable significant GCA for most agronomic traits under the two infestation conditions. Moreover, Line1×Nubaria5, Line2×Sakha3, Line2×Marina, Line3×Nubaria5, Line4×Nubaria1, Line4×Sakha3, Line5×Marina, Line6×Nubaria1 and Line7×Nubaria5 showed desirable SCA for most agronomic traits under both conditions.

The biplot approach is used to analyze combining abilities, heterosis, and parent relationships, providing a graphical representation through principal components (PC1 and PC2) [30]. The biplot has a rapid and graphical presentation of the data, which boosts the ability to recognize patterns of the evaluated data [51]. GGE biplot elucidated most of the variation in the studied traits ranging from 77.35% forweight seed of plot (g) to 90.5% for plant height under *Orobanche*-free soils. These variations were sufficient to assess the stability, and adaptability of lines and testers, and their interaction effects using the GGE biplot. In this respect Yan and Kang [30] depicted that the estimates based on the biplot will be more precise when the biplot explains the high variation. GGE biplot illustrated three elements: (i) the average tester coordinate (ATC) represented by a small circle, indicating the average tester’s position; (ii) the ATC abscissa, depicted as a thick arrowhead line passing through the biplot origin and the ATC pointing from the biplot origin to the average tester, and (iii) the ATC ordinate, a thick double-arrowhead Line perpendicular to the ATC abscissa. The direction and position of these elements assist in defining the GCA effect of an entry concerning the average tester. The polygon derived from the biplot provides insights into line×tester interaction [52].

The polygon is formed by connecting the farthest entries from the origin while keeping all other entries within the polygon. From the origin, lines perpendicular to each side of the polygon are drawn, dividing the biplot into sectors. Testers within the same sector shared a common best-mating partner, identified as the entry at the vertex of the polygon within that sector. This entry is farthest from the origin within its sector [30]. The cross value between the entry and tester is represented by the perpendicular distance between the tester vector Lineextending from the biplot origin to the tester marker position and the entry marker position. Entries positioned at the polygon vertices are the most compatible mating partners with the highest SCA within their sector but may be less compatible with testers in other sectors. Entries closer to the biplot origin are less influenced by changes in testers. The results of GGE biplot analysis are closely aligned with the conventional Kempthorne’s line×tester analysis. These findings concur with previous findings of Ruswandi et al. [37], Badu-Apraku and Akinwale [53, 54], Momeni et al. [55], Oghan et al. [56].

The highly significant variations due to lines×testers displayed different combining ability effects for the assessed lines and testers and were indicative of both additive and non-additive control of the inheritance for the studied traits [21]. Both additive and non-additive gene action have been reported to be preponderance in the inheritance of former characters in faba bean [21, 50, 57, 58]. The prevalence of dominance variances compared to additive variances across all studied agronomic traits signifies the presence of non-additive gene effects. This indicates the potentiality of cross-breeding to generate transgressive offspring. Moreover, such gene actions under line the need to delay the selection of superior plants concerning these traits to later generations, enabling trait improvement by selecting recombinants within segregating populations [22, 42].

## Conclusion

Considerable genetic variations were observed among the parental materials, indicating heterotic effects for all studied agronomic traits. This detected genetic variability and heterotic effects offer potential for improving faba bean performance under both *Orobanche*-free and infested soil conditions. Among the parental genotypes, Line1, Line2, Line3, and Line5 exhibited superior performance under *Orobanche* infestation, displaying the highest values for the studied agronomic traits. Specifically, the parental materials Line3, Line7, and Line2 were notable for reducing the dry weight of *Orobanche* spikes. Additionally, the developed crosses; Line2×Sakha3, Line3×Nubaria5, Line7×Nubaria5, Line6×Nubaria1, Line5×Sakha3, Line1×Sakha3, and Line1×Nubaria5 emerged as the most successful crosses for seed yield and contributing traits under *Orobanche* infestation, demonstrating effectiveness in reducing dry weight of *Orobanche* spikes and enhancing yield traits. These identified crosses present promising sources for achieving high seed yield and tolerance to *Orobanche crenata*. Notably, the results from line×tester analysis using GGE biplot closely aligned with the classical method of Kempthorne’s line×tester analysis.

### Supplementary Information

**Supplementary Material 1.**

## Data Availability

The data are available from the corresponding author upon reasonable request.
