# Supplementary material for: Genetic potential and inheritance pattern of agronomic traits in faba bean under free and infested Orobanche soil conditions
Source: BMC Plant Biol. 2024 Apr 19;24:301. doi: 10.1186/s12870-024-05017-4 (PMC11027393; doi:10.1186/s12870-024-05017-4)
Supplement: Supplementary file 1 — Supplementary Material 1. [file 12870_2024_5017_MOESM1_ESM.docx]

Table S1. The climatic data of the growing seasons 2020-2021 and 2021-2022 at the experimental site

| Month | Temperature (^O^C) | | | | | | Relative humidity (%) | | Rainfall (mm) | |
| --- | --- | --- | --- | --- | --- | --- | --- | --- | --- | --- |
|  | 2020-2021 | | | 2021-2022 | | |  |  |  |  |
|  | Max. | Min. | Mean | Max. | Min. | Mean | 2020-2021 | 2021-2022 | 2020-2021 | 2021-2022 |
| November | 25.00 | 17.50 | 21.25 | 28.1 | 16.7 | 22.40 | 71.80 | 65.8 | 12.35 | 12.7 |
| December | 22.90 | 13.70 | 18.30 | 20.1 | 11.5 | 15.80 | 71.70 | 70.5 | 18.78 | 26.7 |
| January | 21.00 | 13.50 | 17.25 | 17.0 | 7.4 | 12.20 | 73.10 | 72.7 | 51.90 | 50.35 |
| February | 21.19 | 12.50 | 16.85 | 20.1 | 8.7 | 14.40 | 71.70 | 63.4 | 14.65 | 25.25 |
| March | 22.34 | 14.61 | 18.48 | 20.7 | 9.0 | 14.85 | 66.10 | 60.3 | 5.40 | 5.25 |
| April | 28.50 | 19.45 | 23.98 | 31.0 | 13.7 | 22.35 | 60.10 | 51.9 | 0.0 | 0.0 |

Table S2: The soil properties of the experimental site

| **Characteristics** | **2021** | **2022** |
| --- | --- | --- |
| EC dS/m/ 25 C^0*^ | 2.36 | 2.32 |
| Ca^++^ (meq L^-1^) | 4.95 | 4.86 |
| Mg^++^ (meq L^-1^) | 2.83 | 2.78 |
| Na^+^ (meq L^-1^) | 16.02 | 15.75 |
| K^+^  (meq L^-1^) | 0.17 | 0.18 |
| Co_3_^--^ (meq L^-1^) | 0 | 0 |
| Hco_3_^-^ (meq L^-1^) | 3.63 | 3.15 |
| Cl^-^ (meq L^-1^) | 11.21 | 11.01 |
| SO_4_^--^  (meq L^-1^) | 9.15 | 9.41 |
| SAR | 9.68 | 9.6 |
| Soil pH ^**^ | 8.21 | 8.09 |
| Organic matter (%) | 1.17 | 1.18 |
| Available N (mg/kg) | 43.31 | 44.87 |
| Available P (mg/kg) | 9.34 | 8.98 |
| Available K (mg/kg) | 323 | 287 |
| **Available micro-elements (mg/kg)** | | |
| Fe | 43.4 | 42.5 |
| Zn | 8.52 | 8.61 |
| Mn | 30.5 | 30.7 |
| Cd | 0.24 | 0.26 |
| Pb | 1.75 | 1.73 |
| Texture | Clayey | Clayey |
| FC (%) | 45.12 | 45.24 |
| PWP (%) | 24.18 | 24.42 |
| Bulk density (Mg/m^3^) | 1.35 | 1.34 |
| Total porosity (%) | 49.06 | 49.43 |
| IR (cm/hr) | 0.95 | 0.96 |
| PR (N/cm^2^) | 230 | 220 |
| CaCo_3_ (%) | 2.35 | 2.37 |

*: measured in soil paste extract

Soil samples were taken from the experimental sites from the surface layer (0-25 cm) before planting for chemical and physical analysis.

pH = soil reaction (in 1:2.5 soil: water suspension), EC= (Soil salinity) Electrical conductivity (in soil paste extract), ESP = Exchangeable sodium percentage; PR: soil penetration resistance, soluble cations and anions (meq/L soil), ESP: exchangeable sodium percentage FC: field capacity; PWP: permanent wilting point
